# Pupillometry of Groove: Evidence for Noradrenergic Arousal in the Link Between Music and Movement

**DOI:** 10.3389/fnins.2018.01039

**Published:** 2019-01-10

**Authors:** Daniel L. Bowling, Pablo Graf Ancochea, Michael J. Hove, W. Tecumseh Fitch

**Affiliations:** ^1^Department of Cognitive Biology, University of Vienna, Vienna, Austria; ^2^Department of Psychological Science, Fitchburg State University, Fitchburg, MA, United States

**Keywords:** music, rhythm, movement, groove, arousal, pupil

## Abstract

The capacity to entrain motor action to rhythmic auditory stimulation is highly developed in humans and extremely limited in our closest relatives. An important aspect of auditory-motor entrainment is that not all forms of rhythmic stimulation motivate movement to the same degree. This variation is captured by the concept of musical groove: high-groove music stimulates a strong desire for movement, whereas low-groove music does not. Here, we utilize this difference to investigate the neurophysiological basis of our capacity for auditory-motor entrainment. In a series of three experiments we examine pupillary responses to musical stimuli varying in groove. Our results show stronger pupil dilation in response to (1) high- vs. low-groove music, (2) high vs. low spectral content, and (3) syncopated vs. straight drum patterns. We additionally report evidence for consistent sex differences in music-induced pupillary responses, with males exhibiting larger differences between responses, but females exhibiting stronger responses overall. These results imply that the biological link between movement and auditory rhythms in our species is supported by the capacity of high-groove music to stimulate arousal in the central and peripheral nervous system, presumably via highly conserved noradrenergic mechanisms.

## Introduction

The capacity to entrain motor action to rhythmic auditory stimulation is a fundamental characteristic of our species. Widely expressed and flexibly realized in the contexts of music and dance, it is observed across cultures, develops early in life and responds rapidly to training (Darwin, 1871; Nettl, 2000; Phillips-Silver and Trainor, 2005). It is also deeply tied to emotion, as moving to music is associated with feelings ranging from mild pleasure and relaxation to ecstasy and joy (McNeill, 1995; Janata et al., 2012). Recent studies have codified the link between movement and music in terms of “groove” (Janata et al., 2012), defined as the aspect of music that induces a subjective motivation to move along with the beat. The perception of groove varies in response to short musical excerpts and is highly consistent across listeners (Chronbach’s alpha = 0.8–0.9; Madison, 2006; Janata et al., 2012). High-groove is also positively correlated with the accuracy and perceived ease of motor entrainment (Janata et al., 2012; Leow et al., 2014). These findings accord with neuroimaging results showing that coupling between auditory and motor centers of the brain is increased by rhythmic auditory stimulation, even in the absence of actual movement (Popescu et al., 2004; Chen et al., 2006, 2008; Grahn and Rowe, 2009; Fujioka et al., 2012; Lahdelma and Eerola, 2014). This raises the possibility that groove can be systematically manipulated to examine the link between music and movement on a neurophysiological level (Stupacher et al., 2013).

Recent work has emphasized the importance of key acoustic and metrical properties in determining the amount of groove associated with a particular piece of music (Madison et al., 2011; Stupacher et al., 2016). Among acoustic properties, bass (i.e., low-frequency energy) plays a prominent role in transmitting rhythmic information in a variety of genres and styles (Pressing, 2002). Dynamic bass activity is correlated with groove ratings in music, and manipulations that increase bass energy in synthetic drum patterns elicit higher groove ratings (Stupacher et al., 2016). Further, bass appears to shape sensorimotor entrainment behavior, driving the action of large effectors like the hips and feet and supporting synchronization between individuals (Hove et al., 2007, 2014; Van Dyck et al., 2013; Stupacher et al., 2016; Burger et al., 2017; Solberg and Jensenius, 2017; Wojtczak et al., 2017). Finally, a recent EEG study demonstrated that cortical activity corresponding to the perceived beat in music is selectively enhanced when the rhythm is conveyed by low as opposed to high frequency sounds (Lenc et al., 2018).

Among metrical properties, a key role for syncopation in determining groove has long been implicated by its association with high-groove genres such as funk, soul and hip-hop (Greenwald, 2002; Butler, 2006; Danielsen, 2006). Syncopation is a way of systematically violating the rhythmic expectations of a listener (Fitch and Rosenfeld, 2007; Witek et al., 2014a). Theoretical models define it as the occurrence of sonic events at metrically weak (unexpected) locations followed by their omission at metrically strong (expected) locations (e.g., Longuet-Higgins and Lee, 1984). Experimental manipulations of syncopation in synthetic drum stimuli suggest an inverted-U relationship to groove, with moderate levels of syncopation producing the highest levels of groove, and relatively higher/lower levels producing substantially less groove (Sioros et al., 2014; Witek et al., 2014b). Together, these findings suggest bass and syncopation as critical factors that determine groove in music.

Building on this research, we sought to determine whether musical groove is associated with characteristic effects on the nervous system, as well as the extent to which such effects may be dependent on spectral content and syncopation. Neural effects were assessed using pupillometry to measure time-varying pupil diameter, an accessible indicator of arousal in the peripheral and central nervous system (Costa and Rudebeck, 2016; Joshi et al., 2016). In addition to variation in response to light, pupil diameter responds to a variety of cognitive factors (e.g., mental effort, working memory, language processing and pain; Andreassi, 2007), with several recent studies documenting systematic changes in response to music (Gingras et al., 2015; Laeng et al., 2016; Weiss et al., 2016). For example, Gingras et al. (2015) found that pupil dilation in response to short musical excerpts was positively correlated with listener ratings of perceived arousal and tension, and Weiss et al. (2016) found that pupil dilation is greater in response to sung melodies than the same melodies played on a piano. Here, we investigate the relationship between pupil diameter and groove across three experiments. In Experiment 1, we recorded pupil diameter while presenting listeners with excerpts of commercially available high- and low-groove music to test that hypothesis that higher levels of groove elicit higher degrees of physiological arousal in ecologically valid musical stimuli. In Experiment 2, we recorded pupil diameter while presenting listeners with high-pass and low-pass filtered versions of the high-groove music from Experiment 1 to test the hypothesis that low frequency content is particularly effective at stimulating the physiological effects of groove. In Experiment 3, we recorded pupil diameter while presenting listeners with synthetic drum patterns manipulated to vary bass content and amount of syncopation. This allowed us to test the hypothesis that bass content and syncopation play key roles in the stimulating effects of groove under rigorously controlled conditions.

Finally, equal numbers of male and female participants were included in each experiment, allowing us to test for sex differences in physiological reactions to groove. A clear divide in the debate over the evolutionary origins of music is between theories based on social cohesion and theories based on courtship. Social cohesion theories rationalize our capacity to entrain motor action to rhythmic auditory stimulation by referencing the fact that musically inspired motor coordination between individuals tends to increase prosociality (reviewed in Rennung and Göritz, 2016), which is in turn hypothesized to have provided individuals in more musical groups of our ancestors with a competitive edge over those in less musical groups (Roederer, 1984; McNeill, 1995; Freeman, 2001; Dunbar, 2004). Courtship theories rationalize the same capacity via sexual selection, proposing that females preferred to mate with males demonstrating exceptional skill in rhythmic auditory-motor coordination during courtship displays (Darwin, 1871; Miller, 2001; Merker et al., 2009). Sex differences related to auditory-motor entrainment are predicted by courtship theory but not social cohesion theory.

## Materials and Methods

### Subjects

Thirty-two adults (16 males and 16 females; mean age = 24, range = 20–32) participated in this study in exchange for monetary compensation. Individuals had taken weekly music and/or dance lessons for an average of 5.4 years (*SD* = 4.9), and amount of musical training was not significantly different between males and females (Mann–Whitney *U* = 84.5, *p* = 0.3927). All subjects were naïve to the research questions. The experiments were approved by the University of Vienna Ethics Committee (Protocol #00063) and all subjects gave written informed consent in accordance with the Declaration of Helsinki.

### Apparatus

Pupillometric data was collected using an SR Research EyeLink 1000 head-supported infrared optical eye-tracking system. All stimuli were presented over Beyerdynamic DT-770 Pro 80 Ohm headphones (5 Hz–35 KHz frequency response) connected to an Apple Mac Mini computer (model 4.1) running OSX 10.11.6. A computer monitor was used to display a fixation cross during trials and a countdown during rest periods. Stimulus presentation and the pupillometric recording were controlled by custom Matlab code (version R2015a; Mathworks, 2015) written using functions from the Psychophysics (v3.0.14) and Eyelink Toolbox extensions (Brainard, 1997; Pelli, 1997; Cornelissen et al., 2002).

### Procedure

**FIGURE 1:**
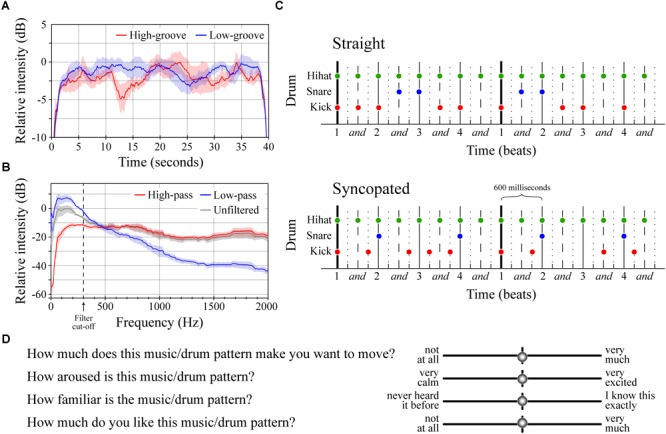
Stimuli and survey questions. **(A)** Average intensity contours for the four low-groove and four high-groove stimuli in Experiment 1. Intensity contours were calculated from .wav files using Praat (function ‘To_intensity’, settings = default; Boersma and Weenink, 2016), smoothed in Matlab using a moving average (function ‘smooth.m’, span = 1 s), and expressed with respect to the maximum intensity of both curves. Shaded error bars represent ± 1 SEM. **(B)** Average spectral envelopes of the four high-pass and four low-pass stimuli from Experiment 2 shown alongside the average spectral envelope of the four unfiltered high-groove stimuli. The average spectrum of each stimulus was calculated by averaging spectra of 100 ms windows running from the beginning to end with 50% overlap in Matlab (functions ‘Hamming.m’ and ‘fft.m’). Envelopes were calculated by smoothing the average stimulus spectrum (‘smooth.m’, span = 200). Shaded error bars represent ± 1 SEM. **(C)** Diagrams of the straight and syncopated drum patterns with circles marking the onset of each drum hit. Vertical lines depict standard metrical divisions for each bar; line style denotes metrical weight: thick > thin > dashed > dotted. **(D)** Survey questions and response entry sliders.

Upon entering the lab, subjects received a brief explanation of the experimental procedure and provided written informed consent. They were then seated in front of the eye-tracker with their heads placed on the chin-rest. The height of the seat was adjusted as required and example stimuli similar to those used in the experiments (see below) were played so that they could adjust the volume to a comfortable level, which was then maintained for the rest of the session (volume levels were not significantly different between males and females; Mann–Whitney *U* = 258, *p =* 0.1480). The three pupillometry experiments were run in two blocks, with an opportunity for a short break in between. Block one consisted of Experiment 3 (drum patterns) and block two consisted of Experiment 1 (music) followed by Experiment 2 (filtered music). The decision to start with the relatively simple drum patterns and end with more dynamic musical stimuli was made to mitigate subject boredom and fatigue in later stages of the experimental session. Each block began with a calibration of the eye-tracking system followed by two practice trials using example stimuli. Trials lasted 50 s: 10 s of silence followed by 40 s of stimulus playback. During trials, subjects were instructed to minimize blinking and focus on a gray fixation cross presented on a black background at the center of the computer monitor. Between trials, a 40-s countdown was presented on the monitor and subjects were encouraged to rest their eyes in preparation for the next trial. After completing the pupillometry experiments, subjects took a brief computerized survey in which they heard each stimulus a second time and provided ratings of groove, arousal, familiarity and preference (Figure 1D). Finally, participants were paid 10 Euros and debriefed. Experimental sessions lasted approximately 45 min.

### Stimuli

The stimuli in Experiment 1 were created from excerpts of commercially available music previously rated for groove in Janata et al. (2012). Eight instrumental excerpts, closely matched for tempo (mean = ∼110 beats per minute), were selected: four with relatively high-groove ratings and four with relatively low-groove ratings (Table 1). Excerpts were obtained by downloading the original track from the iTunes music store (AAC compression, sampling rate = 44100 Hz, bit rate = 260–293 kbps) and extracting the 30-s window used by Janata et al. (2012). Once extracted, each excerpt was extended to 40-s by identifying a time-point within the last 5 s at which the content could be looped without disrupting the beat. One-second linear ramps were then applied to the start and end of each stimulus to create ‘fade-in’ and ‘fade-out’ effects. Finally, the amplitude of each stimulus was adjusted so that all had approximately the same average sound pressure level when played through the DT-770 headphones as measured using a RION NL-62 sound level meter (set to dBA; RION, Tokyo, Japan; Figure 1A). Looping was performed in Logic Pro X (version 10.2.0; (Apple, 2013)). Fades and amplitude adjustments were applied in Matlab and then exported as .wav files for presentation (sampling rate = 44100, bit depth = 16). All subjects heard all eight stimuli in randomized order, making it possible to compare the effects of high- vs. low-groove within-subjects.

**Table 1:** Real music stimuli.

Track name	Artist	Tempo (BPM)	Mean Groove Rating
**High groove stimuli**		**Mean = 110**	**Mean = 90.9**
Sco-mule (DJ Logic remix)	Bernie Worrell, Chris Wood, Gov’t Mule and John Scofield	120	93.9
Bad tune	Earth, Wind and Fire	118	86.2
Outa-space	Billy Preston	115	90.9
Look ka py py	The Meters	87	92.5
**Low groove stimuli**		**Mean = 108.5**	**Mean = 37.3**
Beauty of the sea	The Gabe Dixon Band	120	32.1
Bryter layter	Nick Drake	119	40.4
Druid fluid	Yo-Yo Ma, Mark O’Connor and Edgar Meyer	115	38.1
Ray dawn ballon	Trey Anastasio	80	38.5

*Groove ratings are from Janata et al. (2012), and were collected on a scale from 0 to 127 (Janata et al., 2012); BPM, Beats Per Minute.*

The stimuli in Experiment 2 were filtered versions of the four high-groove stimuli from Experiment 1 (Figure 1B). The filter type, a first-order Butterworth filter, was selected because its relatively gradual roll-off (-6 dB per octave) retained some energy in the stop band, preserving some of the character of the original stimulus but emphasizing spectral content in the pass band. This filter was applied to each original stimulus twice: once as a low-pass filter to create a version emphasizing low-frequency energy, and once as a high-pass filter to create a version emphasizing high-frequency energy. Both applications used a cutoff frequency of 300 Hz. Finally, 1-s linear ramps were applied to the start and end of each stimulus, amplitudes were adjusted to approximately the same mean sound pressure level (dBA), and the results were saved as .wav files following the procedures described above. Filtering was performed in Matlab (functions: ‘butter.m’ and ‘filtfilt.m’). Unlike Experiment 1, Experiment 2 was conducted between-subjects with half of the participants hearing only the low-pass stimuli and the other half hearing only the high-pass stimuli (both groups half male/female). This was done to mitigate potential order effects resulting from repeated exposure to similar stimuli.

The stimuli in Experiment 3 were four synthesized drum patterns designed to cross manipulations of frequency content and metrical structure. Each drum pattern consisted of samples of three drum types (high-hat, snare and kick) from the “Portland Kit” instrument in Logic Pro X. These samples were arranged to create two-bar drum patterns in 4/4 time played at 100 BPM (total duration = 4.8 s). The number of hits on each drum type was the same across patterns. Frequency content was manipulated by altering the pitch of the snare and kick drums (Stupacher et al., 2016). In the ‘low-drums’ patterns, the original drum samples were used (peak frequencies at 40 and 135 Hz respectively). In the ‘high-drums’ patterns, the snare and kick drums were pitch-shifted up using the ‘Inspector > Transpose’ function in Logic Pro X (new peak frequencies at 135 and 228 Hz respectively). The amplitude of the shifted samples was adjusted to achieve comparable loudness with the original samples using the ‘loudness for impulsive sounds’ model in the Loudness Toolbox extension for Matlab (function: ‘Loudness_LMIS.m’; Boullet, 2005). Structure was manipulated by shifting the onset time of snare and kick hits (Figure 1C). In the ‘straight’ patterns, all hits occurred at relatively strong metrical locations resulting in a pattern with no syncopation (polyphonic syncopation score = 0; Witek et al., 2014b). In the ‘syncopated’ patterns, multiple hits occurred at weak metrical locations before omissions at stronger locations, resulting in a level of syncopation near the top of the inverted-U curve described by Witek et al. (2014b; polyphonic syncopation score = 41). These manipulations resulted in four patterns: straight low-drums, straight high-drums, syncopated low-drums, and syncopated high-drums. Each pattern was looped ∼8 times to create a 40 s stimulus with linear fades were applied to first and last second. The results were exported as .wav files for presentation.

### Analysis of Pupil Size

The diameter of the left pupil and the x-y position of the subject’s gaze on the computer monitor were collected continuously throughout each trial (sampling rate = 1000 Hz). This data was processed offline in Matlab to remove artifacts introduced by blinks and visual fixations away from the cross. During blinks, the eye-tracker loses the pupil and records an arbitrary value. These samples were identified and removed including a 50 ms buffer immediately before and after (Gingras et al., 2015). Fixations away from the cross were identified as samples with x-y coordinates located outside a circular region centered on the fixation cross. These samples were also excluded along with a 50 ms buffer immediately before and after. In accordance with other pupillometric studies, the gaps introduced by these procedures were linearly interpolated and the resulting continuous traces were smoothed using a low-pass filter (cutoff = 4 Hz) to remove jitter (Sirois and Brisson, 2014). The smoothed trace from each trial was then baseline-normalized by dividing each sample by the mean calculated over a 1 s baseline immediately preceding stimulus onset. Trials in which more than 50% of the data was excluded (either during baseline or stimulus playback) were excluded from further analyses, resulting in the loss of 25/512 trials (4.8%).

### Statistics

**FIGURE 2:**
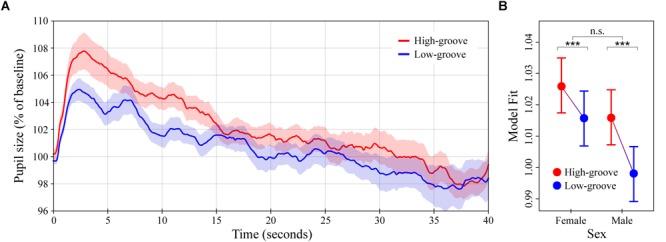
Pupil dilation as a function of groove in music. **(A)** Colored lines depict average baseline normalized pupil diameter over time in response to high- and low-groove music stimuli (*N* = 32 subjects for each curve). Stimulus onset occurred at 0 and lasted until 40 s. **(B)** LME model estimates of baseline normalized pupil diameter showing the significant two-way interaction between groove and sex (see main text). Error bars (shaded and lines) represent ± 1 SEM. ^∗∗∗^*p* < 0.0001; n.s., not significant (Bonferroni corrected).

**FIGURE 3:**
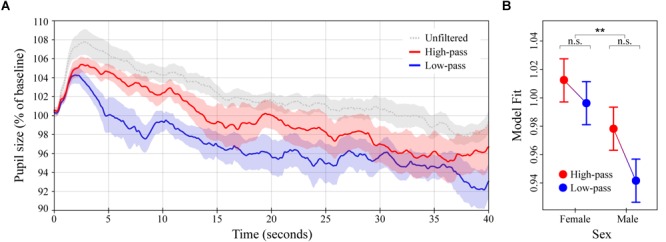
Pupil dilation as a function of filtered high-groove music. **(A)** Colored lines depict average baseline normalized pupil diameter over time in response to high- and low-pass filtered versions of the high-groove stimuli from Experiment 1 (*N* = 16 subjects for each curve). The gray line depicts the response to the original high-groove stimuli (from Experiment 1) for comparison. Stimulus onset occurred at 0 and lasted until 40 s. **(B)** LME model estimates for baseline normalized pupil diameter showing the significant main effect of sex (see main text). Error bars (shaded and lines) represent ± 1 SEM. ^∗∗^*p* < 0.01; n.s., not significant (Bonferroni corrected).

**FIGURE 4:**
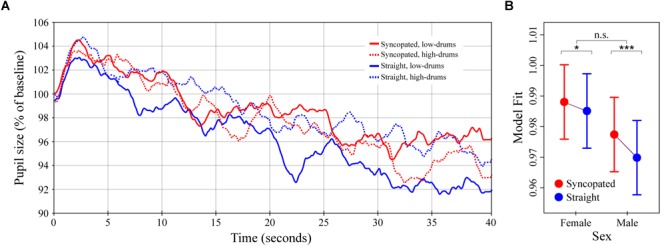
Pupil dilation as a function of metrical structure and frequency content in synthetic drum patterns. **(A)** Colored lines depict average baseline normalized pupil diameter over time in response to the four drum patterns crossing manipulations of metric structure (straight vs. syncopated) and spectral content (high- vs. low-drums; *N* = 32 subjects for each curve). Stimulus onset occurred at 0 s and playback lasting until 40 s. **(B)** The simplified LME model estimates for baseline normalized pupil diameter showing the significant interaction between structure and sex (see main text). Error bars (shaded and lines) represent ± 1 SEM. ^∗^*p* < 0.05; ^∗∗∗^*p* < 0.0001; n.s., not significant (Bonferroni corrected).

Experimental effects on pupil diameter were evaluated for statistical significance in two ways. In the first approach, comparisons were made using standard hypothesis tests (Wilcoxon signed-rank tests in Experiments 1 and 3, Mann-Whitney *U* tests in Experiment 2) to compare distributions of individual subject means calculated over time periods in which pupil diameter was maximally differentiated between experimental conditions (estimated by visual inspection of the average traces in Figures 2–4; Gredebäck and Melinder, 2010; Otero et al., 2011). In the second approach, experimental effects were assessed using linear mixed effects (LME) models to predict baseline-normalized pupil diameter as a function of effects of interest over the full 40 s of stimulation (as represented by averages calculated every 100 ms). These LME models included fixed effects for the experimentally manipulated factors (groove in Experiment 1, spectral content in Experiment 2, metrical structure and spectral content in Experiment 3), listener sex, the interactions between these variables, time since stimulus onset (0–4000 in 100 ms steps), and a random (intercept) effect for subject (ID# 1-32). The same effect structures were also used to model responses to the survey questions. All statistics were performed in RStudio (v1.0.153; R Core Team, 2015). The ‘wilcox.test’ function from package ‘stats’ (v3.3.3) was used to conduct Wilcoxon sign-rank and Mann–Whitney *U* tests (R Core Team, 2012); the ‘lmer’ function from package ‘LME4’ (v1.1-13) was used to build models (Bates et al., 2015); the ‘anova’ function from package ‘car’ (v2.1-5) was used to assess the significance of fixed effects in LME models (Fox et al., 2013); the ‘contrast’ function from package ‘lsmeans’ was used to conduct *post hoc* comparisons for significant interactions (Length, 2016); all *p*-values reported in association with *post hoc* contrasts were corrected using the Bonferroni method; finally, the ‘effects’ function from package ‘effects’ (v4.0-0) was used to plot interactions (Fox, 2003).

## Results

### Experiment 1: High- vs. Low-Groove Music

The average pupillary response to the high- and low-groove music stimuli are shown in Figure 2A. In general, the response to the onset of musical stimulation was characterized by a sharp initial increase in pupil diameter followed by a gradual decline. High-groove stimuli evoked greater dilation than low-groove stimuli on average, with the difference being most pronounced over a time window from 0 to 15 s post onset. Comparison of individual subject means calculated over this period indicated a statistically significant difference, *M*_HighG_ = 104.9% (*SD* = 4.9%) vs. *M*_LowG_ = 102.7% (*SD* = 3.8%), *W* = 149, *p* = 0.0315. Comparison of individual subject means calculated over the remaining 25 s showed greater similarity between responses, *M*_HighG_ = 100.4% (*SD* = 4.9%) vs. *M*_LowG_ = 100.0% (*SD* = 5.7%), *W* = 304, *p* = 0.4545.

The LME model analysis showed similar results. Groove was a significant predictor of pupil dilation, *χ*^2^(1) = 456.6, *p* < 0.0001, with the response to high-groove music estimated to be 0.56% greater than the response to low-groove music on average over the entire 40 s of stimulation (*β* = 0.0056, *SE* = 0.0008). The interaction between groove and listener sex was also a significant predictor, *χ*^2^(1) = 131.0, *p* < 0.0001 (Figure 2B). *Post hoc* analyses showed that although dilation to high-groove music was greater than dilation to low-groove music in both males and females (*ts* = 22.7 and 13.3 respectively, *ps* < 0.0001), the difference was ∼1.6x larger in males, *β*_Male_ = 0.018 vs. *β*_Female_ = 0.011 (*SEs* = 0.00080). Finally, pupil size was predicted to decrease with time post stimulus onset at a rate of ∼0.2% per second, *β* = -0.00019, *SE* = 2.4 ×10^-6^, *χ*^2^(1) = 5599.1, *p* < 0.0001.

### Experiment 2: Filtered High-Groove Music

The average pupillary response to the high- and low-pass filtered versions of the high-groove music stimuli from Experiment 1 are shown in Figure 3A. The overall pattern of pupil dilation was similar to that observed previously, albeit with lower maxima and a slightly more rapid initial decline in response to the low-pass (lower frequency content) stimuli. High-pass stimuli evoked greater dilation than the low-pass stimuli on average, with the difference again being most pronounced over a time window from ∼0 to 15 s post stimulus onset. Comparison of individual subject means calculated over this period indicated a statistically significant difference, *M*_HighP_= 102.8% (*SD* = 3.2%) vs. *M*_LowP_= 99.7% (*SD* = 3.5%), *U* = 205, *p* = 0.0275. As in Experiment 1, comparison of individual subject means calculated over the remaining 25 s showed greater similarity between responses, *M*_HighP_= 97.6% (*SD* = 7.2%) vs. *M*_LowP_= 95.2% (*SD* = 6.1%), *U* = 230, *p* = 0.2067.

The LME model analysis showed similar results. Pupil dilation was estimated to be 1.6% stronger in response to high-pass stimuli than in response to low-pass stimuli over the entire 40 s of stimulation, *β* = 0.016 (*SE* = 0.023). However, because of the reduction in statistical power associated with the between-subjects design, smaller number of subjects per condition, and increased response variance in this experiment, the main effect of spectral content was only marginally significant in this model, *χ*^2^(1) = 2.89, *p* = 0.0890. To address this we recalculated the LME model on the first 15 s post stimulus onset. This resulted in a much higher level of significance for spectral content, *β* = 0.034 (*SE* = 0.016), *χ*^2^(1) = 7.37, *p* = 0.0066, suggesting that pupil dilation was truly differentiated during this early time window. Both versions of the LME model additionally included a significant effect of listener sex, with females exhibiting greater pupil dilation than males overall, *β* = 0.034 (*SE* = 0.023), *χ*^2^(1) = 7.6, *p* = 0.0058 (40-s version; Figure 3B), and *β* = 0.029 (*SE* = 0.016), *χ*^2^(1) = 5.4, *p* = 0.0207 (15-s version). Despite the relationship between spectral content and listener sex resembling that observed between groove and sex in Experiment 1 (cf. Figures 2B, 3B), their interaction was not significant in either model. Finally, over the entire 40 s of stimulation, pupil diameter was predicted to decrease with time post stimulus onset at a rate equal to that found in Experiment 1, *β* = -0.0002/100 ms (*SE* = 3.5 × 10^-6^), *χ*^2^(1) = 4158.3, *p* < 0.0001.

### Experiment 3: Drum Patterns

The average pupillary responses to the drum pattern stimuli are shown in Figure 4A. The general pattern of pupil dilation was similar to that observed in Experiments 1 and 2 albeit with lower maxima and more rapid declines. Unlike Experiments 1 and 2, however, no obvious differences between conditions were apparent in the average responses. Comparison of individual subject means calculated over time windows from 0–15 to 16–40 s post onset likewise failed to show any significant differences.

The results of the LME model analysis informed this absence of simple differences by uncovering a significant three-way interaction between metrical structure, spectral content and listener sex, *χ*^2^(1) = 1279.4, *p* < 0.0001. A detailed exploration of this three-way interaction is presented in Supplementary Text 1 and Supplementary Figure 1. For the present analysis, however, we chose to run a simplified LME model designed to predict pupil dilation as a function of metrical structure, sex, the interaction between these factors, and time (i.e., omitting spectral content and its interactions). The result of the simplified LME model analysis showed a significant effect of metrical structure, with syncopation resulting in 0.3% greater pupil dilation over the entire 40 s of stimulation, *β* = -0.003 (*SE* = 0.0009), *χ*^2^(1) = 61.6, *p* < 0.0001. The interaction between metrical structure and sex was also a significant predictor, *χ*^2^(1) = 11.8, *p* < 0.001 (Figure 4B). *Post hoc* analyses showed that although dilation to the syncopated patterns was greater than dilation to the straight patterns in both males and females (*t*s = 8.0 and 3.1, *ps* < 0.0001 and 0.0108 respectively), the difference was ∼2.5x larger in males than females, *β*_Male_= 0.0075 vs. *β*_Female_= 0.0029 (*SEs* = 0.0009). Finally, in keeping with the results of Experiments 1 and 2, pupil diameter was predicted to decrease with time post stimulus onset at a rate of -0.23% per second, *β* = -0.00023 (*SE* = 3.0 × 10^-6^), *χ*^2^(1) = 5491.2, *p* < 0.0001.

### Survey Results

**FIGURE 5:**
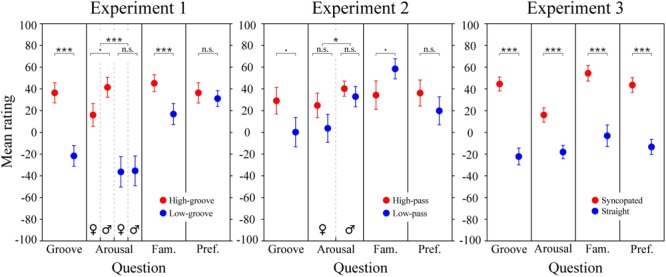
Survey results. Mean groove, arousal, familiarity, and preference ratings for the stimuli in Experiments 1, 2, and 3. Each data point represents an average rating calculated across subjects. Error bars represent ± 1 SEM. Male and female responses are shown separately when the corresponding LME model analysis predicted an effect of sex or an interaction including sex. Statistics for Experiment 3 are from the simplified LME model analysis (see main text); for Experiment 3 ratings and statistics separated by metrical structure and spectral content and statistical results of the full LME model, see Supplementary Figure 2. ^∗∗∗^*p* < 0.0001; ^∗^*p* < 0.05; ^.^*p* < 0.09; n.s., not significant.

The results of the survey are shown in Figure 5. For the stimuli in Experiment 1 (left panel), LME modeling of the survey responses confirmed that the high-groove stimuli selected from Janata et al. (2012) were rated by our participants as higher in groove than the low-groove stimuli, *β* = 49 (*SE* = 8.2), *χ*^2^(1) = 76.4, *p* < 0.0001, as well as more arousing, *β* = 52.4 (*SE* = 8.1), *χ*^2^(1) = 100.7, *p* < 0.0001, and more familiar *β* = 22.0 (*SE* = 7.5), *χ*^2^(1) = 26.9, *p* < 0.0001. Parallel to the pupillary results, a significant interaction was found between groove and listener sex for arousal ratings, *χ*^2^(1) = 4.5, *p* = 0.0339, with post-hoc comparisons showing that males tended to perceive high-groove stimuli as more arousing than females on average, *β* = 25.4 (*SE* = 9.5), *t*(71.3) = 2.67, *p* = 0.0563. Thus, physiological and perceptual measures suggest that responses to high- and low-groove music are more differentiated in males compared to females.

For the stimuli in Experiment 2 (Figure 5, middle panel), LME modeling of the survey responses showed marginally significant effects of spectral content on groove and familiarity ratings. The high-pass stimuli tended to be rated as higher in groove than the low-pass stimuli, *β* = 28.3 (*SE* = 21.2), *χ*^2^(1) = 3.2, *p* = 0.0730, and the low-pass stimuli tended to be rated as more familiar than the high-pass stimuli, *β* = 22.4 (*SE* = 18.5), *χ*^2^(1) = 3.5, *p* = 0.0619. Listener sex had a significant effect on arousal ratings, *β* = 15.1 (*SE* = 15.7), *χ*^2^(1) = 4.2, *p* = 0.0402, but the direction was opposite to that expected based on the pupillary results: males rated the stimuli as more arousing than females despite female exhibiting stronger physiological reactions.

To examine survey responses to the stimuli in Experiment 3 (Figure 5; right panel), we applied the same simplified LME model used to investigate pupil dilation. The results showed a significant effect of metrical structure for all four questions. Ratings of the syncopated patterns were higher for groove, *β* = 66.3 (*SE* = 10.7), arousal *β* = 37.3 (*SE* = 9.1), familiarity, *β* = 54.0 (*SE* = 12.9), and preference, *β* = 59.3 (*SE* = 9.6), *χ*^2^s(1) = 41.9, 21.1, 27.1, and 38.7 respectively, *ps* < 0.0001. Models including spectral content and its interactions showed an additional significant effect, with the low-drum patterns being preferred over high-drum patterns, *β* = 12.0 (*SE* = 11.5), *χ*^2^(1) = 4.5, *p* = 0.0329 (see Supplementary Figure 2).

## Discussion

The experiments reported here show that pupil dilation exhibits clear sensitivity to various manipulations of groove in music. Specifically, we found greater pupil dilation in response to high- vs. low-groove music, high- vs. low-pass filtered music, and syncopated vs. straight drum patterns, as well as greater differential sensitivity among males despite stronger overall reactions among females. These results are largely consistent with previous research based on surveys as well as self-reported evaluations during our experiment, extending them by providing evidence of physiological sensitivity. Major exceptions include the more stimulating effects of high- compared to low-frequency spectral content, and the robust and consistent sex differences, both of which appear to be novel.

In Experiment 1, we compared the effects of high- vs. low-groove music, finding that high-groove music stimulated stronger reactions than low-groove music. This difference developed rapidly, increasing to almost 3% after several seconds and maintaining around 2% for nearly 15 s. Despite the high- and low-groove responses being more similar during the remaining 25 s of stimulation, the LME model analysis still estimated a difference of ∼0.6% over the entire 40 s trial. Importantly, this effect is not explained by simple differences in the tempo or overall loudness of the stimuli, both of which were controlled between conditions. Comparison of the average intensity contours of the high- and low-groove stimuli further confirms that local intensity differences (e.g., during the first 15 s) do not account for these effects (see Figure 1A). An interesting aspect of the pupillary responses in Experiment 1 is their dependence on the sex of the listener. Although both male and female pupils dilated more in response to high-groove music than low-groove music, the difference was estimated to be ∼1.6x larger in males (see Figure 2B), raising the possibility that males exhibit greater physiological sensitivity to differences in musical groove.

The survey results were generally consistent with the physiological results. In addition to stimulating greater pupil dilation, high-groove music was perceived as more associated with a desire to move, more arousing and more familiar (although no subjects were familiar enough with the individual tracks to name them or the artists that created them). Intriguingly, ratings of arousal were determined to depend on listener sex in parallel with pupil dilation. The difference in average ratings between the high- and low-groove stimuli was ∼1.5x larger for males than females (see Figure 5, left panel). In sum, the results of Experiment 1 demonstrate that high-groove music stimulates higher levels of physiological arousal than low-groove music, and suggests that males may exhibit greater differential sensitivity to groove than females.

In Experiment 2, we compared the effects of high- vs. low-pass filtered high-groove music. Given the common association between groove and bass (see Introduction), this experiment was designed to probe the relative effects of low- and high-frequency spectral content on physiological arousal. Contrary to our expectations, we found greater pupil dilation in response to high-pass stimuli than the low-pass stimuli over the first 15 s of stimulation, suggesting that high-frequency content plays a critical role in the stimulating effects of high-groove music. As in Experiment 1, this effect is unlikely to be explained by differences in the tempo or loudness of the stimuli, which were controlled between conditions. Also in parallel with Experiment 1, the amount of pupil dilation observed in Experiment 2 depended on the sex of the listener. The relationship between spectral content and sex closely resembled that between groove and sex in Experiment 1, with females exhibiting greater pupil dilation overall, but males exhibiting greater differential sensitivity (cf. Figures 2B, 3B). However, only the main effect of listener sex was significant in the LME model analysis (15- and 40-s versions). A final point about the pupil data is that both filtered versions of the stimuli in Experiment 2 resulted in less dilation than their original (unfiltered) counterparts. This is consistent with the commonsense notion that full-spectrum music is more stimulating than partial-spectrum music. However, we cannot fully attribute this difference to an effect of spectral content because subjects heard the stimuli in a fixed order (full-spectrum first), which could have decreased responsiveness to the partial-spectrum stimuli via habituation. Some evidence against this possibility comes from a recent study in which repeated presentation of the same musical stimulus increased rather than decreased pupil dilation, as observed here (Weiss et al., 2016).

Insight into the surprising physiological effects of spectral content comes from the survey results. Subjects tended to perceive the high-pass stimuli as higher in groove than the low-pass stimuli, and perceived the low-pass stimuli as more familiar than the high-pass stimuli. During debriefing, multiple subjects explained these ratings by stating that the low-pass stimuli sounded like the muffled sound heard standing outside a nightclub, or when the neighbor is playing loud music. This suggests that, compared to the high-pass stimuli, the low-pass stimuli lost more of their identifying characteristics (e.g., melodic and rhythmic information carried in higher- frequency bands) and thus sounded more generic. Intriguingly, ratings of arousal were again determined to depend on listener sex. However, unlike in Experiment 1, the direction was opposite to that observed in pupil dilation (males rated the stimuli as more arousing than females despite exhibiting less dilation). In sum, the results of Experiment 2 demonstrate a critical role for high-frequency spectral content in the stimulating effects of high-groove music. Together with previous work this suggests a dual account of spectral content in groove, with low-frequency content inducing auditory-motor entrainment, and high-frequency content stimulating and maintaining physiological arousal. At the level of musical phenomenology, this accords with the fact that drum-kits and rhythm sections typically pair low frequency drums with higher frequency idiophones (bells, cymbals, etc.), the later often played at more rapid sub-beat intervals.

In Experiment 3, we examined the effects of systematically manipulating metrical structure and spectral content in synthetic drum patterns. Modeling the results of this experiment resulted in a significant three-way interaction between these factors and listener sex (see Supplementary Figure 1). Given the difficulties inherent in interpreting this type of interaction, as well as the limited conclusions that can be drawn from them, we decided to drop spectral content from the analysis and construct a simplified LME model focused on metrical structure and sex. Our rationale for this adjustment was twofold. First, the fact that the high- but not low-drum patterns contained spectrally manipulated (pitch-shifted) samples introduced a potential confound. Second, the results of the survey suggested that metrical structure played the more important role in determining responses. The simplified LME model showed a clear effect of metrical structure, with greater pupil dilation in response to the syncopated patterns compared to the straight patterns. This provides compelling evidence for a role of syncopation in the physiologically stimulating effects of groove and is consistent with perceptual studies relating groove and syncopation (Witek et al., 2014b) as well as the actual use of syncopation to enhance groove in music (Pressing, 2002). In parallel with the sex differences observed in Experiments 1 and 2, we found evidence of greater differential sensitivity to syncopation in males compared to females, with the difference in male responses between the straight and syncopated patterns being ∼2.5x greater than the difference in female responses on average.

The survey results for Experiment 3 were consistent with the physiological results with the exception that they failed to show any interactions. Syncopation resulted in higher ratings across all survey questions but did not interact with spectral content or listener sex on any question. In summary, the results of Experiment 3 provide evidence for physiological sensitivity to metrical structure, with syncopation producing larger effects in males than females.

Although there is substantial evidence that rhythm perception engages auditory and motor regions of the brain, this is the first study to demonstrate differences in pupil dilation associated with differences in groove. Recent studies have established a firm link between pupil dilation and activity in the locus coeruleus (LC), a pontine nucleus that constitutes the largest group of noradrenergic neurons in the central nervous system (Samuels and Szabadi, 2008). Using electrophysiological methods in rhesus macaques, Joshi et al. (2016) demonstrated that activity in the LC is closely correlated with pupil dilation in spontaneous and stimulus-driven contexts, and that electrical stimulation in the LC evokes pupil dilation more consistently than stimulation in other regions (e.g., the inferior and superior colliculi; Costa and Rudebeck, 2016; Joshi et al., 2016). Combined with the current study these results suggest that high levels of groove may activate the LC, which would increase the release of noradrenalin (norepinephrine) at multiple sites. Noradrenergic projections from the LC play a critical role in the regulation of arousal and autonomic function and could underpin music’s link to movement in a number of distinct ways. These include: (1) increasing cortical activity and responsiveness through the LC’s direct innervation of all lobes of the cerebral hemispheres, the basal forebrain and thalamus (Berridge and Foote, 1991; Berridge et al., 1993); (2) increasing heart rate and blood pressure through subcortical projections to the hypothalamus, brainstem and spinal chord; (3) facilitating motor planning and coordination through excitatory projections to the cerebellum; (4) increasing skeletal muscle tone through projections to the ventral horn of the spinal chord; and (5) increasing the sensitivity of acoustic processing mechanisms through excitatory projections to the cochlear nuclei (Ebert, 1996; Samuels and Szabadi, 2008). To the extent that groove activates the LC, the link between music and movement can thus be hypothesized to build upon a number of distinct mechanisms tired to noradrenalin, many of which are likely to be shared across species (Sharma et al., 2010).

The sex differences found in this study are of particular interest with respect to ongoing debates over the evolution of human musical behavior. Although female pupillary reactions were stronger overall, male pupillary reactions differed more as a function of groove, spectral content and metrical structure. Although physiological differences between males and female reactions to music have been documented in a handful of previous studies (McFarland and Kadish, 1991; Altenmüller et al., 2002; Koelsch et al., 2003a,b; Nater et al., 2006; Gingras et al., 2015; Belfi et al., 2016; Laeng et al., 2016), to our knowledge these results constitute the first evidence of sex differences specific to the link between music and movement. Given the culturally limited nature of our sample, the biological implications of such differences are limited. Nevertheless, we briefly discuss their potential relevance to current hypotheses about the adaptive functions of music (cf. Fitch, 2006). The sex differences we observed here are parsimoniously accommodated by courtship theory because sexual selection often results in dimorphic traits (Lande, 1980). From this perspective, the greater differential sensitivity of males would specifically enhance performance in the context of high-groove music, where differences in coordination skill between males are likely to be most apparent. Females would then use the resulting displays to evaluate candidate mates, choosing only the most skilled. The relatively strong and less differentiated female response would facilitate judgement across groove levels, maximizing the potential for extracting biologically relevant information whenever present. Accommodating sex differences within social cohesion theory is also possible but requires additional assumptions, such as the fitness benefits of group musical activity being conferred mostly through increased prosociality between males. A third possibility is that courtship played a role in initiating the evolution of the motor control systems required for rhythmic coordination, which later came to serve non-sexual functions in other social contexts including strengthening social bonds and potentially other functions like facilitating mother-infant interaction (Dissanayake, 2001). Although it is not possible to differentiate between these alternatives here, our data suggest that further exploration of sex differences is an important line of investigation. Explicitly including sex in the design and analysis of future studies is thus indicated. For example, by measuring whether physiological sensitivity to groove in females increases around ovulation (Charlton et al., 2012; Charlton, 2014), or testing for sex differences in musically inspired prosociality.

Finally, in addition to the empirical findings reported here, this work establishes a physiological correlate of the link between music and movement in our species. Because pupil dilation is relatively easy to measure, this methodology can be extended across cultures, life stages and species in a straightforward manner to assess the extent to which the physiological correlates of groove may be a function of culture, development and phylogeny, providing further insight into the biological foundations of human musicality.

## Conclusion

The experiments reported here provide the first empirical demonstration that music strongly associated with movement (“high-groove” music) stimulates stronger pupil dilation than music weakly associated with movement (“low-groove” music). This result is consistent with the hypothesis that hearing groovy music makes us want to move in part by up-regulating noradrenergic arousal in the central and peripheral nervous system. At a more detailed level, our results show that high-frequency spectra are relatively more effective at stimulating pupil dilation than low-frequency spectra. This contrasts with the wide-spread notion that low-frequencies (“bass”) hold the key to groove, instead suggesting a dual account of spectra in musically inspired movement with complementary roles for low- and high-frequency content in inducing entrainment and stimulating arousal. We also showed that syncopated rhythms stimulate stronger pupil dilation than straight rhythms, providing physiological evidence for the stimulating effects of metrical manipulation in groove. Last but not least, we found consistent sex differences across all three experiments that, if confirmed and extended by further studies, may have important implications for debates concerning the evolution of music and the human capacity for auditory-motor entrainment.

## Author Contributions

DB designed the research, programmed the task, analyzed the data, and wrote the manuscript. PGA collected and analyzed the data and wrote the manuscript. MH designed the research and edited the manuscript. WTF provided resources and supervision, and edited the manuscript.

## Conflict of Interest Statement

The authors declare that the research was conducted in the absence of any commercial or financial relationships that could be construed as a potential conflict of interest.
