# Supplementary material for: Pupillometry of Groove: Evidence for Noradrenergic Arousal in the Link Between Music and Movement
Source: Front Neurosci. 2019 Jan 10;12:1039. doi: 10.3389/fnins.2018.01039 (PMC6335267; doi:10.3389/fnins.2018.01039)
Supplement: Supplementary file 1 [file Data_Sheet_1.docx]

**SUPPLEMENTARY MATERIAL**

**Contents**

**• Supplementary Text 1. Experiment 3 Full LME model Three-Way Interaction**

**• Supplementary Figure 1. Experiment 3 Full LME model Three-Way Interaction**

**• Supplementary Figure 2. Experiment 3 Survey Results Assessed by Full LME model**

**Supplementary Text 1. Experiment 3 Full LME model Three-Way Interaction.** The full LME model analysis of Experiment 3 revealed a significant 3-way interaction between metrical structure, spectral content, and listener sex. Post-hoc analyses of this interaction indicated that males and females reacted differently to structure depending on spectra (Supplementary Figure 1A). The largest difference occurred between males responding to the straight and syncopated low-drum patterns, with the model estimating 4.7% greater dilation in response to the syncopated pattern on average, *β*=0.047 (*SE*=0.0013), *t*(47561.2)=35.9, *p*<0.0001. Females showed an opposite but much smaller difference, with the model estimating 0.47% less dilation in response to the syncopated pattern on average, *β*=-0.0047 (*SE*=0.0015), *t*(47567.6)=-3.2, p=0.0381. Both of these differences were reversed in response to the high-drum patterns, with males responding more strongly to the straight pattern, *β*=-0.033 (*SE*=0.0013), *t*(47563)=-47.34, p < 0.0001, and females responding more strongly to the syncopated pattern, *β*=0.014 (*SE*=0.0014), *t*(47563.2)=10.08, *p*<0.0001. Finally, in keeping with the results of Experiments 1 and 2, pupil diameter was predicted to decrease with time post stimulus onset at a rate of -0.24% per second, *β*=-0.00024 (*SE* = 3.0x10^-6^), *χ*^2^(1)=5820.5, p<0.0001.

Although considerably more complex, the results of the full LME model accord with those of the simplified LME model presented in the main text. In the context of low-drum patterns, pupil dilation was increased in response to syncopation in males but not females, and this pattern was reversed for the high-drum patterns. However, the fact that the difference between responses to the straight and syncopated patterns was larger in the spectral content condition in which syncopation resulted in greater dilation (low-drums for males, high-drums for females) accounts for the significantly stronger effect of syncopation in the simplified model. The full and simplified LME models were also in accord with respect to the effect of sex. In both cases, males exhibited greater differential sensitivity despite females reacting more strongly overall.

With respect to the apparent reversal in the effects of syncopation for high-drum patterns, closer inspection shows that this was primarily the result of differences in pupil dilation in response to the straight pattern (see Supplementary Figure 1A); by comparison spectral content had relatively minor effects on pupil dilation in response to the syncopated pattern. Comparing the effects of spectral content across Experiment 2 and 3 shows a similar pattern for males (greater pupil dilation in response to high-pass and high-drums respectively) but a different pattern for females (greater pupil dilation in response to high-pass but less pupil dilation in response to high-drums), highlighting the fact that the manipulations of spectral content in Experiments 2 and 3 are not equivalent.

**
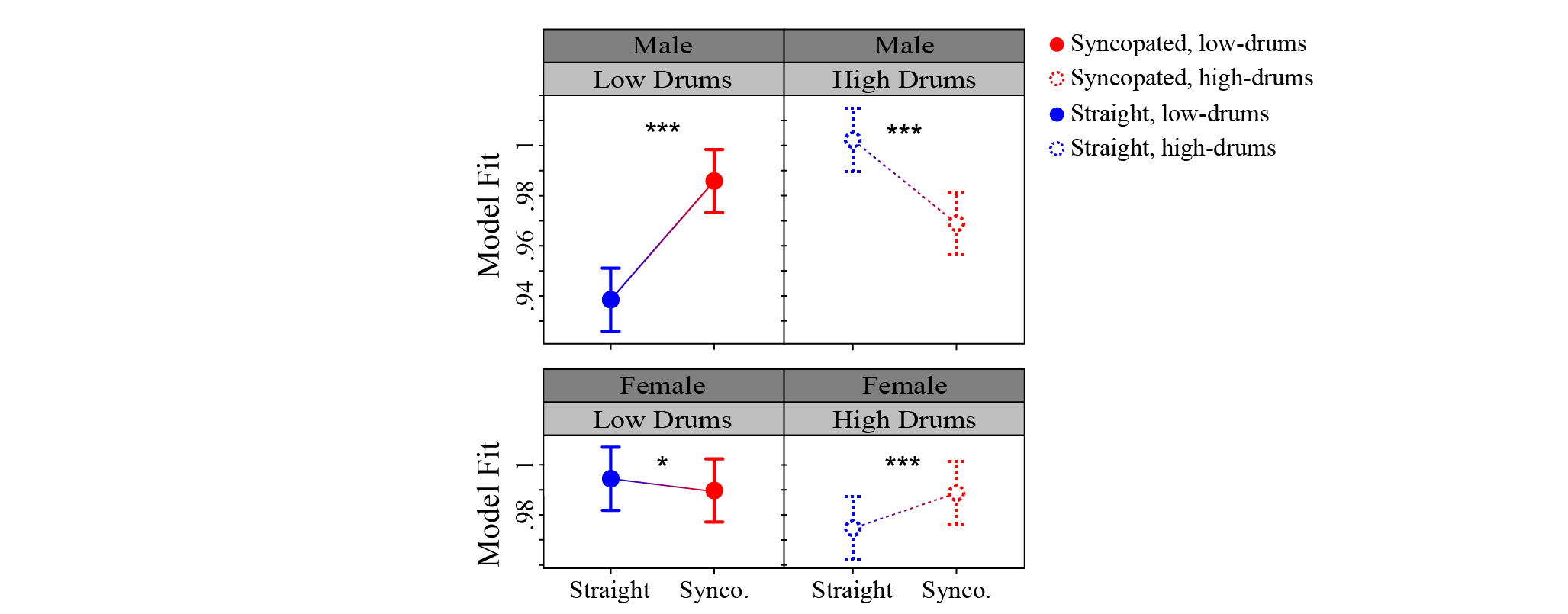
**

**Supplementary Figure 1. Experiment 3 Full LME model Three-Way Interaction.** LME model predictions for baseline normalized pupil diameter showing the significant three-way interaction between metrical structure, spectral content and listener sex (see Supplementary Text 1). Error bars (shaded and lines) represent ±1 SEM. * = p < 0.05; *** = p < 0.0001 (Bonferroni corrected).


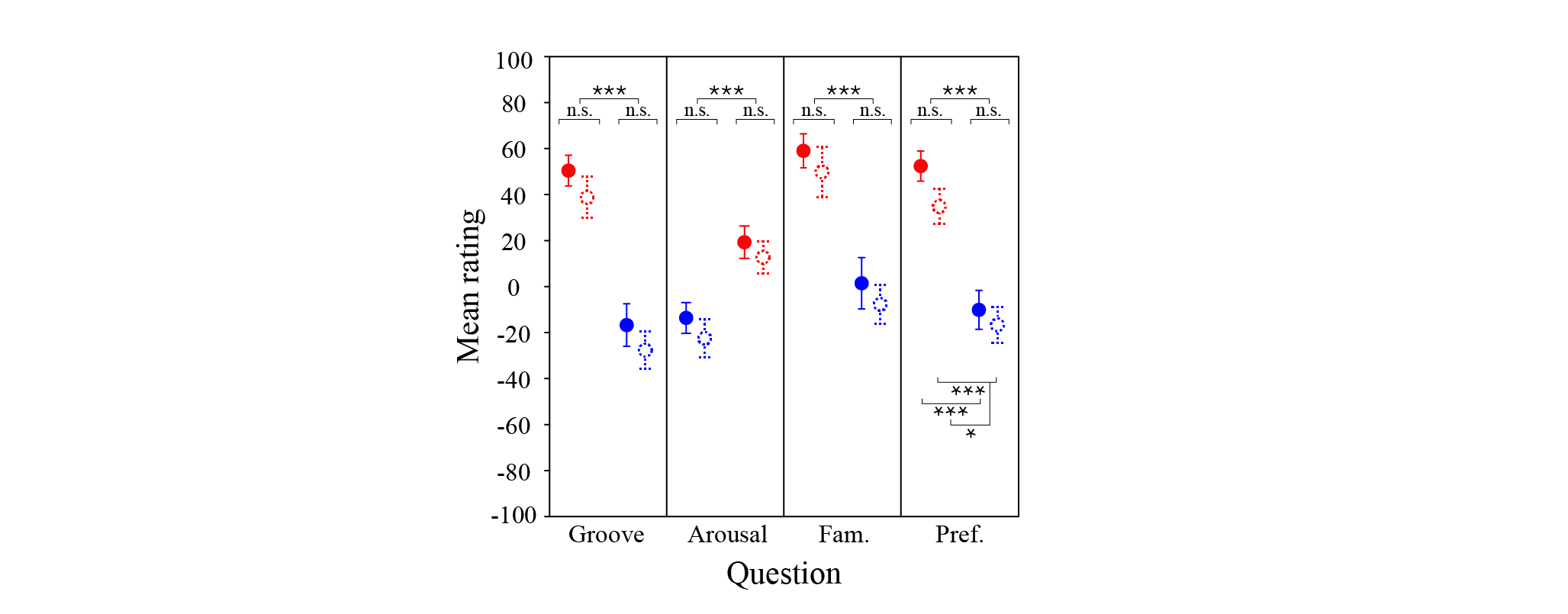


**Supplementary Figure 2. Experiment 3 Survey Results Assessed by Full LME Model**. Mean groove, arousal, familiarity, and preference ratings for the stimuli in Experiments 3 shown separately for low-drum and high-drum patterns. Each data point represents an average rating calculated across subjects. Statistical results are derived from applying the full LME model to the survey ratings (i.e., including spectral content and its interactions). These results are much the same as those derived using the simplified model presented in the main text. Ratings of the syncopated patterns were higher for groove, *β*=63.5 (*SE*=13.3), arousal *β*=41.4 (*SE*=11.0), familiarity, *β*=49.3 (*SE*=14.1), and preference, *β*=47.1 (*SE*=11.1), *χ*^2^s(1)=72.5, 34.2, 53.3 and 70.8 respectively, *ps*<0.0001). Additionally, ratings of low-drum patterns were higher in preference *β*=12.0 (*SE*=11.5), *χ*^2^(1)=4.5, *p*=0.0329.
